# Dysbiosis of the Urinary Microbiota Associated With Urine Levels of Proinflammatory Chemokine Interleukin-8 in Female Type 2 Diabetic Patients

**DOI:** 10.3389/fimmu.2017.01032

**Published:** 2017-08-25

**Authors:** Zongxin Ling, Fengping Liu, Li Shao, Yiwen Cheng, Lanjuan Li

**Affiliations:** ^1^Collaborative Innovation Center for Diagnosis and Treatment of Infectious Diseases, State Key Laboratory for Diagnosis and Treatment of Infectious Diseases, The First Affiliated Hospital, College of Medicine, Zhejiang University, Hangzhou, China; ^2^Nursing School, Jiangsu Vocational College of Medicine, Yancheng, China

**Keywords:** *Akkermansia*, interleukin-8, *Lactobacillus*, type 2 diabetes mellitus, urinary microbiota

## Abstract

Evidence has shown that dysbiosis of the urinary microbiota existed in female type 2 diabetes mellitus (T2DM) patients. Perturbations of intestinal microbiota are linked to proinflammatory chemokine interleukin-8 (IL-8); however, the correlations between urinary microbiota and IL-8 are not well studied. Here, we investigated the associations between the altered urinary microbiota and urinary IL-8 in female T2DM patients. A modified four-tube midstream urine technique was used to collect urine specimens from 70 female T2DM patients and 70 matched healthy controls (HCs). Bacterial genomic DNA from urine specimens was isolated using magnetic beads and the urinary microbiota was assessed using Illumina MiSeq platform targeting on the 16S rRNA gene V3–V4 region. Urinary IL-8 was determined by enzyme linked immunosorbent assay. Subsequently, the T2DM patients were separated into urine IL-8 detectable (WIL8) and undetectable (NIL8) groups, and the composition of urinary microbiota between the two groups was compared. Meanwhile, the levels of IL-8 between the “≥HCs” group (those specific bacterial genera were more than or equal to the HCs) and the “<HCs” group (those specific bacterial genera were less than the HCs) was also compared. Of 70 urine samples from T2DM patients without urinary tract infections, 46 patients had detectable IL-8 in their urine (64.31 ± 70.43 pg/mL), while 24 patients had undetectable IL-8. Compared to the NIL8 group, 11 bacterial genera increased in the WIL8 group, including *Corynebacterium, Akkermansia, Enterococcus*, etc., whereas 10 genera, such as *Faecalibacterium, Bacteroides*, and *Pseudomonas* decreased. One species of *Lactobacillus, Lactobacillus iners*, increased obviously in the WIL8 group. The “≥HCs” group showed 17 genera increased and 16 genera decreased. In addition, 18 genera contributed to the presence of urinary IL-8 in T2DM patients, which explained 95.60% of the total variance of urinary microbiota. Our study demonstrated that dysbiosis of the urinary microbiota with several key bacteria was associated with urinary IL-8 in female T2DM patients, which might be useful to explore the interactions between urinary microbiota and inflammatory responses and shed light on novel diagnosis and therapy for urinary microbiota associated with infections in T2DM patients.

## Introduction

Diabetes mellitus (DM) is a common, serious, and costly disease, which is a major public health issue (1). In recent decades, the global prevalence of DM has increased from 4.7% in 1980 to 8.5% in 2014, while the number of people with diabetes has risen from 108 million in 1980 to 422 million in 2014 (1). In 2012, 1.5 million deaths were directly caused by DM, which has been projected as the seventh leading cause of death in 2030 (1). A previous study has reported that type 2 diabetes mellitus (T2DM) comprises the majority of the people with DM around the world, approximately 90% of DM patients are T2DM patients (2).

Patients with DM are prone to various infections, with the urinary tract as the most common infection site (3, 4). Urinary tract infection (UTI) in hospitalized DM patients was nearly two times higher than that caused by other factors (5). The probability of UTIs in T2DM patients was 60% higher than that in non-DM individuals. A survey showed that the incidence of UTIs in T2DM patients was 46.9 per 1,000 person-years, compared with 29.9 for non-T2DM subjects (6).

Cytokines are small, soluble proteins produced by various cells in response to infections and inflammation (7). Interleukin (IL)-8, a potent proinflammatory chemokine and activator of neutrophils, can be stimulated by lipopolysaccharide, IL-1, and tumor necrosis factor alpha (TNF-α). Previous studies have found that interleukin-8 (IL-8) causes migration of neutrophils to the place of inflammation, leading to pyuria in patients with UTIs (8, 9). Elevated urine levels of IL-8 were detected in febrile children with UTIs compared to children with asymptomatic bacteriuria (7). It is reported that urinary IL-8 can be identified as a potential novel biomarker for the diagnosing of UTIs with 93% sensitivity and 90% specificity (10, 11). Ko et al. found that confirmation of the presence of bioactive IL-8 in urine suggests the participation of IL-8 in UTI, providing additional evidence of the role of IL-8 in inflammation (12). Consequently, the measurement of urinary IL-8 is thought to be a potential bioindicator of the localization and severity of inflammation within the urinary tract (9). Epithelial cell lines secrete interleukin in response to stimulation with bacteria (13). Therefore, it is possible that urinary microbiota modulate the presence and levels of IL-8 in the urinary tract.

Previous studies show that the production of cytokines is related to human microbiota. For instance, Schueller et al. found that Veillonellaceae and Neisseriaceae are the most abundant indicators for high IL-8 status, whereas Erysipelotrichaceae is the most abundant bacteria in low IL-8 subjects in an oral microbiota study (14). One study that explores the cytokine secretion of vaginal epithelial cells induced by commensal bacteria shows that colonization of parallel multilayer cultures with *Staphylococcus epidermidis* resulted in a significant increase in IL-8 relative to non-colonized cultures (15). Cervicovagina those dominated by *Gardnerella* and *Prevotella* induced higher levels of IL-8, whereas *Lactobacillus iners* induced moderate IL-8 secretion and *Lactobacillus crispatus* did not elicit IL-8 secretion (16). On the other hand, IL-8 in combination with other cytokines plays crucial roles in regulating the microbiota. Previous studies have shown that IL-6 and IL-10 play essential roles for the maintenance of intestinal homeostasis and the prevention of colitis (17, 18), while proinflammatory cytokines, such as IL-17 and IL-15 promote intestinal dysbiosis associated with increased susceptibility to colitis (19, 20). In addition, levels of IL-α, IL-β, and IL-6 are able to stimulate the expression of antimicrobial peptides, which help to shape the skin microbiota and have a protective role against potential pathogen attack (21).

Seventy female T2DM patients were recruited in our previous study, since female patients are known to have higher prevalence of UTI than males (22). We explored whether dysbiosis of urinary microbiota was related to T2DM females. We found that both diversity and richness of urinary microbiota declined in T2DM patients. *Actinobacteria, Flavobacteriales*, and *Flavobacteria* were recognized as potential distinguishing biomarkers for T2DM patients. Fourteen bacterial genera, including *Lactobacillus, Prevotella*, and *Streptococcus*, were enriched in the T2DM patients, while 19 bacterial genera, such as *Pseudomonas, Klebsiella*, and *Akkermansia*, decreased. Meanwhile, the relative abundance of *Actinobacteria, Lactobacillus*, and *Akkermansia muciniphila* correlated to the levels of fasting blood and urine glucose (23). The correlations between cytokines and microbiota from intestine, oral cavity, vagina, and skin have been extensively investigated, while the relationship between IL-8 and urinary microbiota remains poorly studied. Here, we investigated whether dysbiosis of urinary microbiota was associated with the presence of urinary IL-8, which might be useful to explore the interactions between the urinary microbiota and immune system and shed light on potential novel diagnosis and therapy for UTIs in T2DM patients.

## Materials and Methods

### Recruitment of Subjects

We used an individually matched case–control design in our previous study, with one control for each T2DM patient. The matching attributes were age in years (based on decade) and marital and menstrual status. In this study, 70 female T2DM patients and 70 healthy controls (HCs) were recruited from the First Affiliated Hospital, School of Medicine, Zhejiang University from June 28, 2015 to January 2, 2016. Both groups ranged from 26 to 85 years old. The body mass index (BMI) in the HCs was 23.10 ± 4.49 kg/m^2^ and in the T2DM group was 23.87 ± 3.65 kg/m^2^. Subjects with the following attributes were excluded: UTIs in the previous month; use of antibiotics, probiotics, prebiotics, or synbiotics in the previous 3 months; unable to complete the questionnaire; menstruation; urinary incontinence; known anatomic urinary tract abnormalities (e.g., cystoceles, hydronephrosis, renal atrophy, or neurogenic bladder); urinary catheter (23). The Ethics Committee of the First Affiliated Hospital, School of Medicine, Zhejiang University approved the study (reference number: 295). Written informed consent based on the Declaration of Helsinki was obtained from each patient before enrollment, and all patients were given informed consent.

### Collection of Urinary Specimens and DNA Sequencing

A modified four-tube midstream urine collection technique was used for the first urine of the day, which guaranteed that the real midstream urine was collected. A urine sample was discarded when it was confirmed to be contaminated. Samples were given anonymous identification codes and were transferred immediately to the laboratory and stored at −80°C until DNA extraction. For urinary microbiota analysis, total DNA was extracted from the pellet of urine from tubes 2 and 3, and 40 mL of urine was aspirated from each tube, separated into three sections, and injected into three 15 mL sterile centrifuge tubes. Each tube was pelleted by centrifugation at 4,000 × *g* for 15 min at 4°C. 10 mL of the supernatant was decanted, and the pellet was obtained by centrifugation for 15 min at 4,000 × *g* at 4°C. The pellet was transferred into a 2 mL sterile centrifugation tube which contained 500 µL of lysis buffer. Magnetic bead isolation of genomic DNA from bacteria was applied according to the manufacturer’s protocol with minor modifications (Supplementary Material: Protocol of DNA Isolation). The 16S rRNA gene V3–V4 regions were amplified from microbial genomic DNA (forward primer, 5'–ACTCCTACGGGAGGCAGCAG–3'; reverse primer, 5'–GGACTACHVGGGTWTCTAAT–3') (23). Urinary IL-8 was determined by enzyme-linked immunosorbent assay kits (RayBiotech, Inc., Norcross, GA, USA), following the manufacturer’s instructions. All measurements were performed in duplicate wells. The lower limit of detection for each assay was 1 pg/mL. Standard curves were generated for every plate and the average 0 standard optical densities were subtracted from the rest of the standards, controls and samples to obtain a corrected concentration.

### Bioinformatic Analysis

Sequencing reads were processed using QIIME (version 1.9.0), and included additional quality trimming, demultiplexing, and taxonomic assignments. KW rank sum test and pairwise Wilcoxon test were used for the identification of the different markers, and LDA was used to score each feature in the LEfSe analysis. An index of alpha diversity was calculated with QIIME based on sequence similarity at 97%. Beta diversity was measured by unweighted UniFrac distance, which was also calculated by QIIME. Diversity and richness of bacteria in the urine specimens were calculated using several estimates. These consisted of the level of operational taxonomic units (which provides a measure of bacterial richness), Chao1 (which is also an estimate of bacteria richness) and the Shannon and Simpson indices (which are measures of bacterial diversity). The output file was further analyzed using Statistical Analysis of Metagenomic Profiles software package (version 2.1.3) (24). Sequence data from this study are deposited in the GenBank Sequence Read Archive with accession number SRP 087709.

### Grouping

From the concentration of urinary IL-8, the T2DM patients were separated into “with IL-8” (WIL8) group, indicating there was detectable IL-8 in their urine; and “no IL-8” (NIL8) group, indicating there was no detectable IL-8 in their urine. Our previous study showed that the relative abundance of 33 bacterial genera in urine were significantly different between the HCs and the T2DM patients (Figure S1 in Supplementary Material) (23). After obtaining the results of differences in relative abundance of bacterial genera in urine between the HCs and T2DM patients (*p* < 0.05) (23), the patients were divided into two groups: “≥HCs” group, indicating relative abundance of bacterial genera were not less than the HCs; and “<HCs” group, indicating the relative abundance of bacterial genera were less than the HCs. Thereafter, the levels of IL-8 in the “≥HCs” group and “<HCs” group were compared.

### Statistical Analysis

Statistical analysis was performed using the SPSS data analysis program (version 21.0) and Statistical Analysis of Metagenomic Profiles software. For continuous variables, independent *t*-test, Welch’s *t*-test, and White’s non-parametric *t*-test were applied. For categorical variables between groups, either the Pearson chi-square or Fisher’s exact test was used depending on assumption validity. For taxon among subgroups, ANOVA test was applied (Tukey–Kramer was used in *Post hoc* test, effect size was Eta-squared) with Benjamini–Hochberg FDP false discovery rate correction (25, 26). All tests of significance were two-sided, and *p* < 0.05, or corrected *p* < 0.05, was considered statistically significant. In addition, stepwise multiple linear regression analysis was used to determine the factors that significantly affected urine microbiota in T2DM patients. All potential variables (*p* < 0.05) were entered into the analysis.

## Results

### Characteristics of T2DM Patients in NIL8 and WIL8 Groups

There were significant differences in age, BMI, urine pH, urine white blood cells, urine leukocyte esterase, urine protein, urine glucose, and urine nitrite between T2DM patients with detectable IL-8 in their urine (WIL8) and those without detectable urine IL-8 (NIL8) (*p* < 0.05, Table 1).

**Table 1:** Descriptive data of participants.

Parameter	Value for cohort (*n*^a^)^b^ or statistic		*p*-Value^c^
	NIL8 (*n* = 24)	WIL8 (*n* = 46)	
Age (years)	59.25 ± 11.06	65.78 ± 13.58	0.046
Duration of type 2 diabetes mellitus (T2DM)	7.63 ± 5.67	10.89 ± 8.11	0.083
UTIs in the last year	0.57 ± 1.20	0.70 ± 1.11	0.656
Body mass index (kg/m^2^)	22.46 ± 3.26	24.61 ± 3.65	0.018
Menstrual status [no. (%)]			0.955
Premenopausal	19 (79.17)	38 (90.94)	
Postmenopausal	3 (12.50)	6 (13.04)	
Hysterectomy	2 (8.33)	3 (6.52)	
Fasting blood glucose (mmol/L)	7.81 ± 2.33	7.84 ± 2.39	0.959
Urine pH	5.58 ± 0.55	5.92 ± 0.63	0.028
Urine white blood cells	2.27 ± 2.93	11.38 ± 24.25	0.015
Urine leucocyte esterase [no. (%)]			0.000
Negative	23 (95.83)	34 (73.91)	
Positive	1 (4.17)	12 (26.09)	
Urine protein [no. (%)]			0.000
Negative	23 (95.83)	33 (71.74)	
Positive	1 (4.17)	13 (28.26)	
Urine culture [no. (%)]			0.094
Negative	24 (100.00)	41 (89.13)	
Positive	0 (0.00)	5 (10.87)	
Urine glucose [no. (%)]			0.000
Negative	21 (87.50)	35 (79.09)	
Positive	3 (12.50)	11 (23.91)	
Urine nitrite [no. (%)]			0.000
Negative	24 (100.00)	40 (86.97)	
Positive	0 (0.00)	6 (13.04)	

*NIL8, no interleukin-8 (IL-8) detected in urine samples from T2DM patients; WIL8, IL-8 detected in urine samples; UTIs, urinary tract infections*.

*^a^No. of subjects*.

*^b^Values are mean ± SD or no. (%)*.

*^c^Pearson’s chi-square and Fisher’s exact tests were used with categorical variables. Independent t-test was used with continuous variables*.

### Sequencing Data and Concentrations of Urinary IL-8

Briefly, we obtained 3,981,519 reads for microbiota analysis, which accounted for 76.93% of the valid reads. The mean read length was 438 bp (range 423–486 bp). The Good’s coverage estimator was 98% (23).

**Figure 1:**
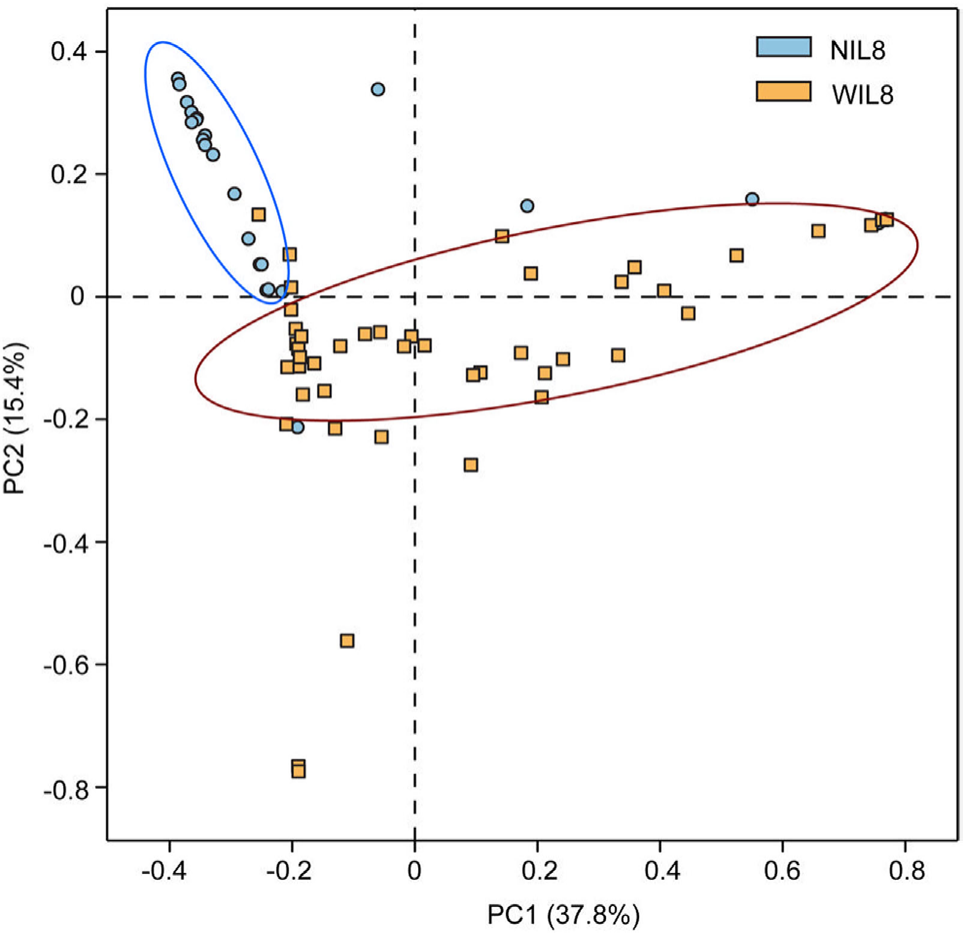
Principal coordinate analysis (PCoA) plot. PCoA plot of the urinary microbiota based on the unweighted UniFrac metric. Blue and yellow dots represent NIL8 and WIL8 specimens, respectively.

The average urinary IL-8 concentration in the 70 patients was 42.26 ± 64.66 pg/mL. Urinary IL-8 was found in 46 samples and concentration was 64.31 ± 70.43 pg/mL. No significant difference was found in Shannon and Simpson indices between the NIL8 and WIL8 group (*p* > 0.05, Table S1 in Supplementary Material). However, principal coordinate analysis (PCoA) indicated that most of the samples from NIL8 and WIL8 groups could be clustered together (Figure 1).

### Urinary IL-8 Associated Biomarkers

**Figure 2:**
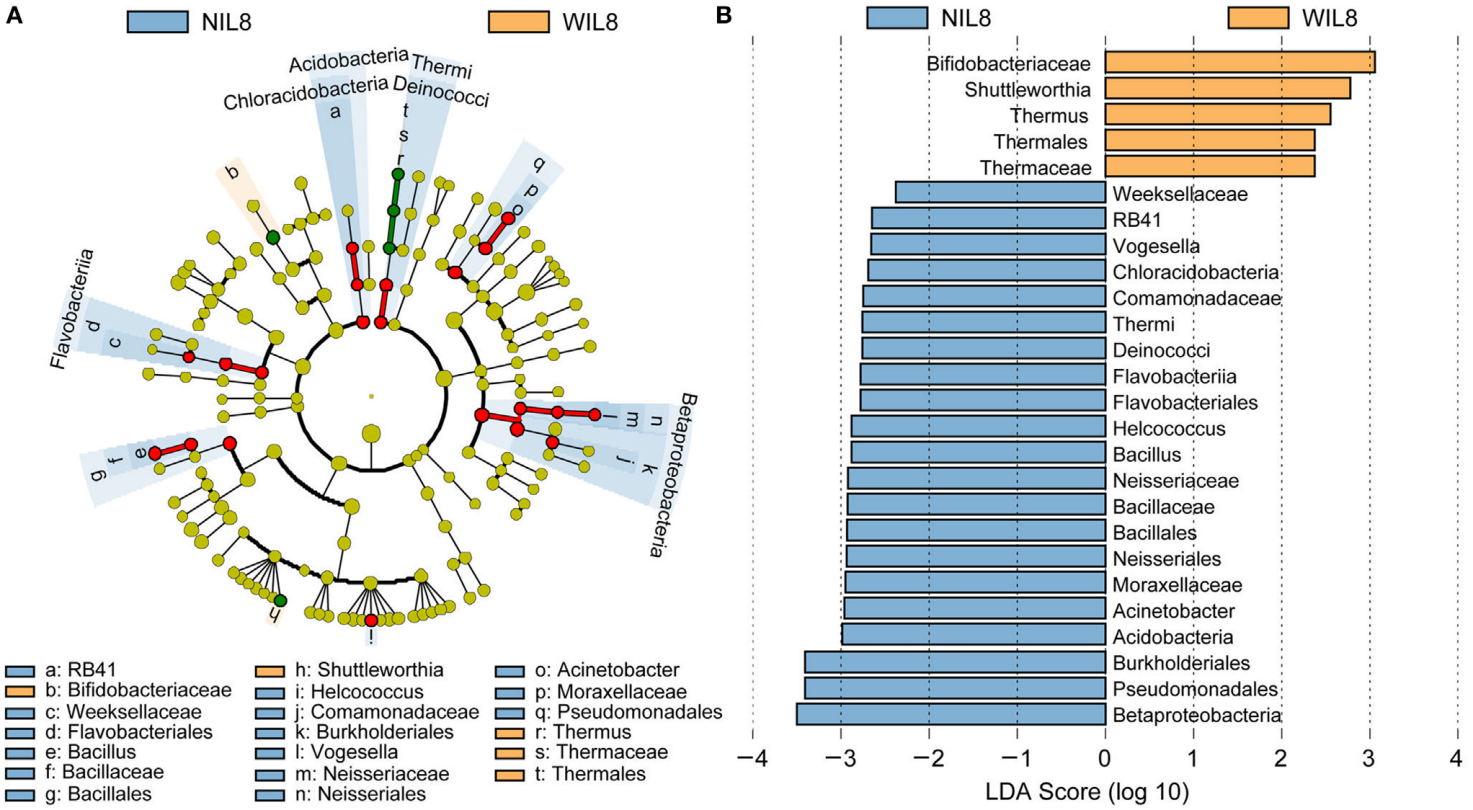
Cladogram showing differentially abundant taxa of urinary microbiota in type 2 diabetes mellitus patients. **(A)** LEfSe cladogram showed the most differentially abundant taxa between WIL8 and NIL8 groups. Taxonomic cladogram obtained from LEfSe analysis of 16S rDNA sequences. Taxa enriched for WIL8 in yellow; NIL8 enriched taxa in blue. The size of each dot is proportional to its effect size. **(B)** Only taxa meeting an LDA threshold > 2.0 are shown.

To identify the specific bacterial taxa associated with urinary IL-8, the urinary microbiota in the WIL8 and NIL8 groups were compared using LEfSe. A cladogram representative of the structure of the urinary microbiota and their predominant bacteria is shown in Figure 2. The greatest differences in taxa between two groups are displayed. Bifidobacteriaceae, *Shuttleworthia, Thermus*, Thermales, and Thermaceae could be used as potential distinguishing biomarkers between the WIL8 and NIL8 groups.

### Associations between Urinary Microbiota and IL-8

**Figure 3:**
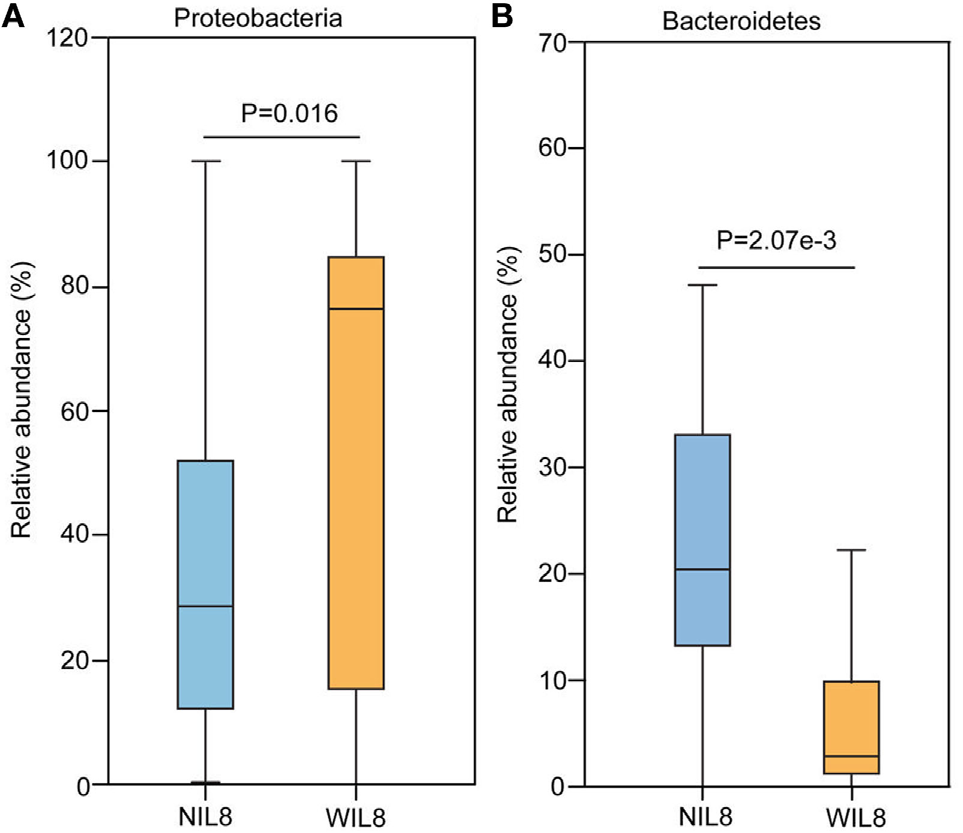
Phylum-level operational taxonomic units different between NIL8 and WIL8 groups. STAMP software was used to calculate the bacterial phylum proportions in the two groups: Proteobacteria **(A)** and Bacteroidetes **(B)**. Welch’s *t*-test was used to compare abundance at the bacterial phylum level for NIL8 urine samples and WIL8 samples. The different phyla were assigned only to those presenting a minimum variation at a significant level [*p* (corrected) < 0.05].

**Figure 4:**
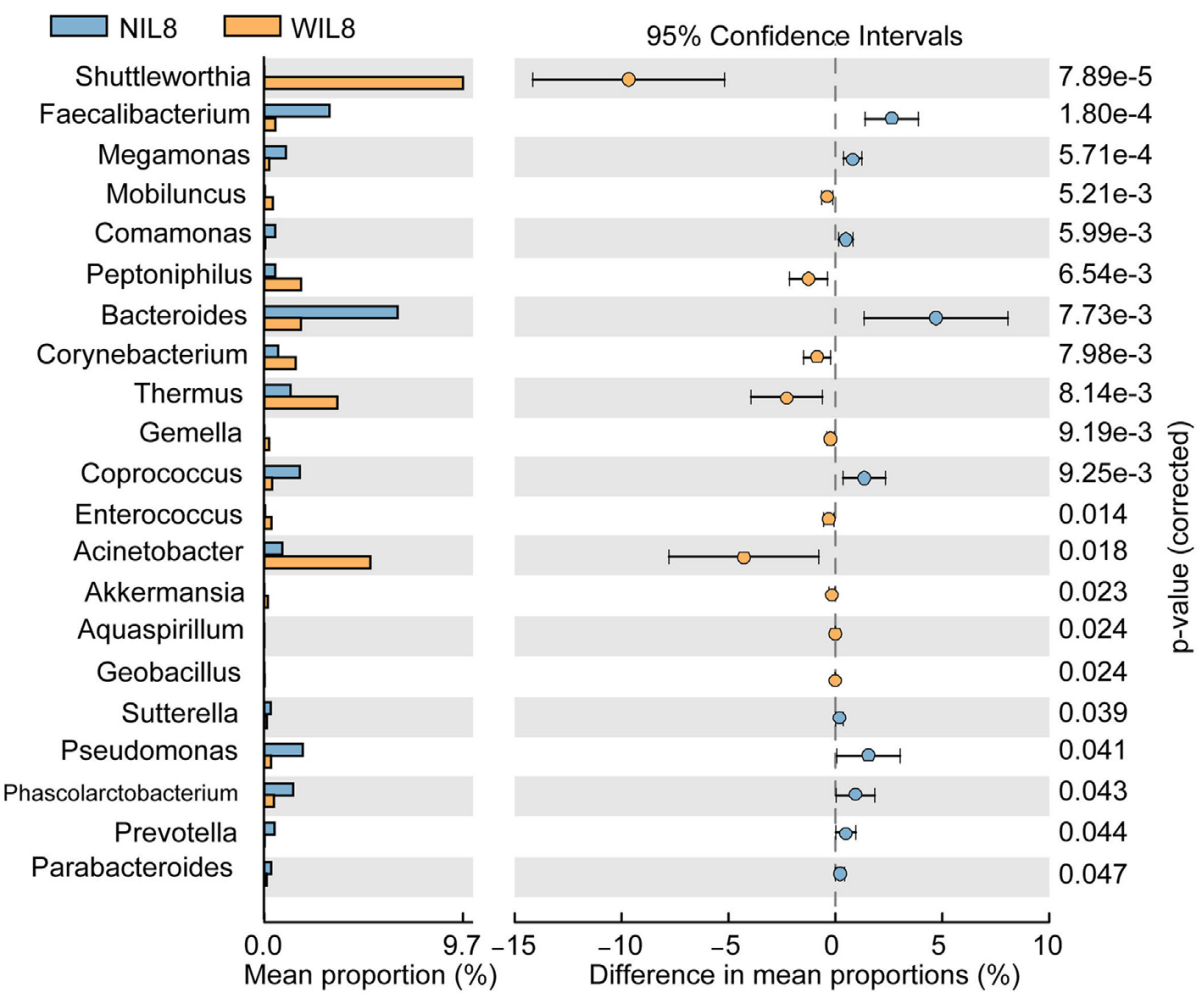
Genus-level operational taxonomic units different between NIL8 and WIL8 groups. STAMP software was used to calculate the genus proportions in the two groups. Welch’s *t*-test was used to compare abundance at the genus level for NIL8 and WIL8 specimens. The different genera were assigned only to those presenting a minimum variation at a significant level [*p* (corrected) < 0.05].

At the phylum level, *Proteobacteria* was significantly higher in the WIL8 group than the NIL8 group, while Bacteroidetes was dramatically decreased in the WIL8 group (*p* < 0.05, Figure 3). At the genus level, 11 genera were enriched in the WIL8 group compared to the NIL8 group, including *Shuttleworthia, Mobiluncus, Peptoniphilus, Corynebacterium, Thermus, Gemella, Enterococcus, Acinetobacter, Akkermansia, Aquaspirillum*, and *Geobacillus*, while 10 genera were decreased, including *Faecalibacterium, Megamonas, Comamonas, Bacteroides, Coprococcus, Sutterella, Pseudomonas, Phascolarctobacterium, Prevotella*, and *Parabacteroides* (*p* < 0.05, Figure 4). Five bacterial species, *Streptococcus anginosus, Acinetobacter rhizosphaerae, Acinetobacter schindleri, L. iners*, and *A. muciniphila*, showed a significant increase in the WIL8 group than the NIL8 group, while another five species showed a significant decrease, including *Prevotella copri, Faecalibacterium prausnitzii, Prevotella stercorea*, and *Bacteroides uniformis*, and *Coprococcus eutactus* (*p* < 0.05, Figure S2 in Supplementary Material). *L. iners* dramatically increased in the WIL8 group. In addition, the *Lactobacillus* species including *Lactobacillus mucosae* and *Lactobacillus reuteri* showed a trend increase in the WIL8 group, whereas *Lactobacillus ruminis* showed a trend decrease, but these species did not reach statistical differences (Figure S3 in Supplementary Material).

**Figure 5:**
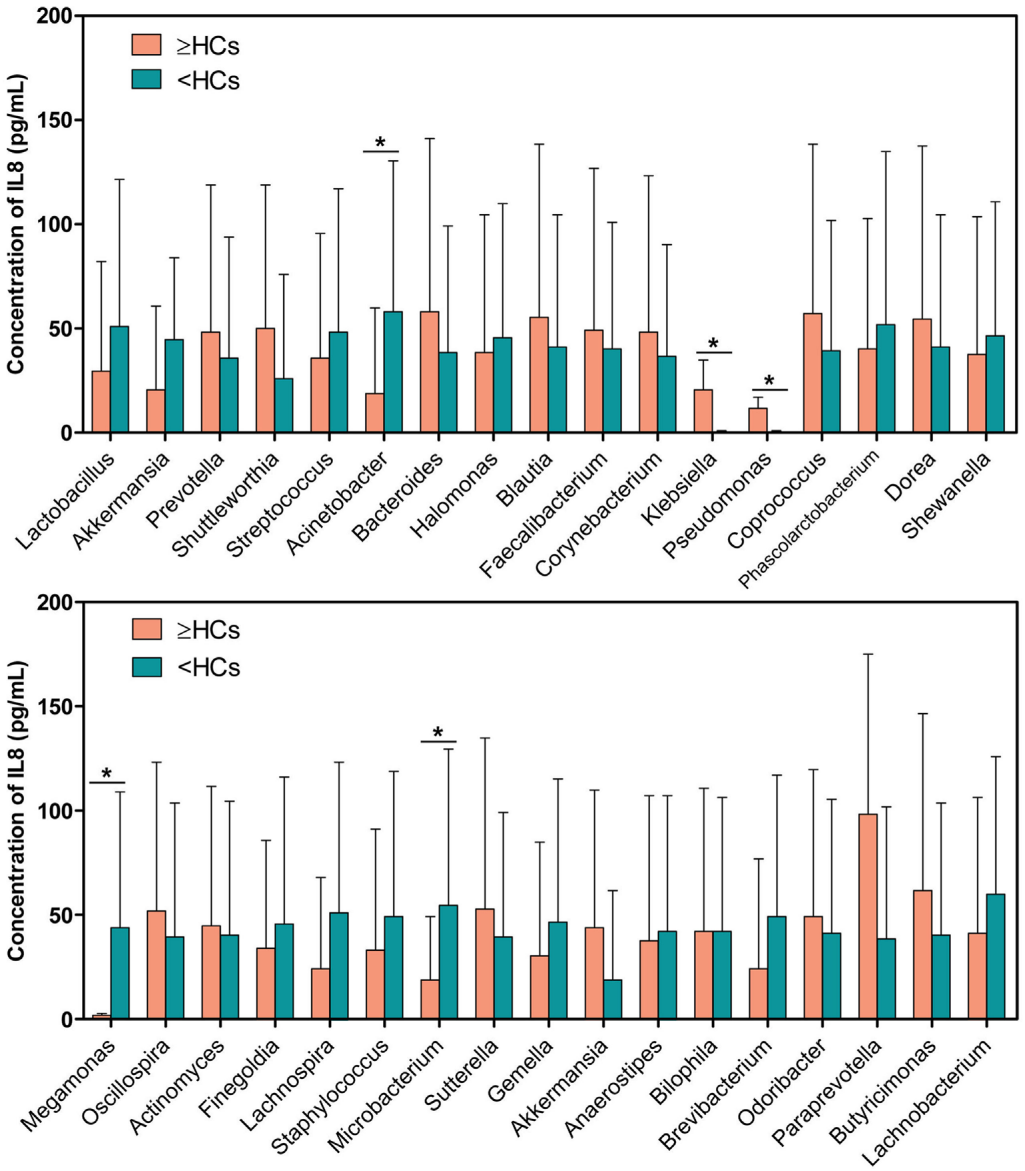
Comparison of interleukin-8 (IL-8) levels between genus “≥HCs” group and “<HCs” group. SPSS software was used to calculate the difference in IL-8 concentrations between bacterial genus “≥HCs” group and “<HCs” group. “≥HCs” group indicates the relative abundance of a bacterial genus in this group was not less than the healthy controls (HCs), and “<HCs” group indicates relative abundance of a bacterial genus was less than the HCs. Asterisks indicate significant differences between the two groups (*p* < 0.05).

Interestingly, 17 bacterial genera were enriched in the “≥HCs” group, while 16 genera were enriched in the “<HCs” group. Specifically, those patients with *Bacteroides* “≥HCs” group, *Klebsiella* “≥HCs” group, *Pseudomonas* “≥HCs” group and *Akkermansia* “≥HCs” group had higher concentrations of urinary IL-8, while those patients with *Lactobacillus* “<HCs” group, *Megamonas* “<HCs” group and *Microbacterium* “<HCs” group had higher levels of IL-8 (Figure 5).

Multiple regression analysis showed that the genera *Ruminococcus, Limnohabitans, Cytophaga, Providencia, Anaerotruncus, Giesbergeria, Solitalea, Actinomyces, Meiothermus, Luteibacter, Flavisolibacter, Dysgonomonas, Ureaplasma, Exiguobacterium, Zoogloea, Cloacibacterium, Lactobacillus*, and *Dokdonella* significantly affected urinary microbiota in T2DM patients and explained 95.60% of the total variance of urinary microbiota in this population (Table 2). In addition, the relative abundance of *Ruminococcus* was significantly positively associated with the levels of urinary IL-8 (Figure S4 in Supplementary Material).

**Table 2:** Predictors of urine IL-8 by stepwise regression (*n* = 70, where *n* is number of patients).

Independent variables	Unstandardized coefficient		Standardized coefficient	*t*	*p*-Value	*F*	*p*-Value
	*B*	SE	β				
Constant	7.610	3.204		2.375	0.021	57.478	0.000
*Ruminococcus*	9.051	0.749	0.380	12.085	0.000		
*Limnohabitans*	4242.295	353.48	0.360	12.002	0.000		
*Cytophaga*	4204.23	330.817	0.481	12.709	0.000		
*Providencia*	13214.688	1239.622	0.319	10.66	0.000		
*Anaerotruncus*	800.311	71.947	0.333	11.124	0.000		
*Giesbergeria*	296.428	29.784	0.355	9.953	0.000		
*Solitalea*	849.692	74.052	0.352	11.474	0.000		
*Actinomyces*	24.412	4.197	0.205	5.816	0.000		
*Meiothermus*	20.241	2.496	0.249	8.110	0.000		
*Luteibacter*	2307.744	347.269	0.199	6.645	0.000		
*Flavisolibacter*	−105.620	24.71	−0.155	−4.274	0.000		
*Dysgonomonas*	5080.332	1180.355	0.144	4.304	0.000		
*Ureaplasma*	13.178	2.615	0.162	5.039	0.000		
*Exiguobacterium*	−258.281	67.919	−0.15	−3.803	0.000		
*Zoogloea*	−3614.663	807.044	−0.147	−4.479	0.000		
*Cloacibacterium*	−76.561	23.057	−0.105	−3.320	0.002		
*Lactobacillus*	0.304	0.092	0.107	3.292	0.002		
*Dokdonella*	−1478.647	617.393	−0.075	−2.395	0.020		

## Discussion

Type 2 diabetes mellitus patients are prone to a higher occurrence of certain infections compared with the healthy population (27). The hospitalization rate for UTIs caused by diabetes is over twice those caused by other factors (5). Defects in maintaining the integrity of mucosal barriers can result in systemic endotoxemia that contributes to chronic low-grade inflammation (28). Recent advances in understanding the pathophysiology of T2DM have established the involvement of low-grade inflammation due to an increased production of proinflammatory cytokines (29, 30). This study focused on the correlations between urinary microbiota and the proinflammatory chemokine IL-8 for the first time, with the aim of providing new insights on host–urinary microbiota interactions in T2DM patients.

Urine specimens from 46 T2DM patients had detectable levels of IL-8 (WIL8 group), while IL-8 was not detected in 24 T2DM patients (NIL8 group). The alpha diversity indices, such as Shannon and Simpson, did not show significant differences between the NIL8 and WIL8 groups, indicating that bacterial diversity was not affected by the presence of IL-8 in urine. The beta diversity index such as PCoA analysis indicated that most of the patients with urinary IL-8 formed their own cluster, which reflected a contribution from IL-8 was prominent in differentiating urinary microbiota in groups.

Interestingly, the distinguishing biomarker, Bifidobacteriaceae, which is associated with diabetic patients with higher BMIs (31), had a higher abundance in WIL8 subjects, and those patients also had higher BMIs than NIL8 participants in our study. Elfeky et al. reported that exosomes increased IL-8 release from endothelial cells, and the effect was even higher when exosomes were isolated from obese women compared to lean subjects (32). Thus, BMI might play a role in regulating IL-8 levels, and subsequently IL-8 modulates the abundance of urinary Bifidobacteriaceae.

A higher abundance of *Proteobacteria* was found in the WIL8 group in this study. Fricke et al. and Rani et al. have demonstrated that *Proteobacteria* in urine decreased in patients that had renal transplantation and were exposed to high dose immunosuppressant (33, 34), during which cytokine production might be inhibited by the suppressing T cells induced by administrating the immunosuppressant. In addition, a recent study on intestinal microbiota reported that *Proteobacteria* was detected in feces of rats at the peak of experimental autoimmune encephalomyelitis (35). Demmer et al. demonstrated that inflammation explained 30–98% of the observed associations between levels of microbiota in a subgingival microbiome study, and the percentages of the overall phyla associations with inflammation were 27% for *Proteobacteria* (36). The above findings illustrated that *Proteobacteria* might be correlated to the onset or development of the inflammatory process. It is demonstrated that a rise in species belonging to the phylum *Proteobacteria* may have a larger impact on host autoimmunity which may make a protein molecule non-functional and thereby may be involved in the onset of inflammatory disorders, including diabetes (37).

The relative abundance of Bacteroidetes was lowered in the WIL8 group. A similar alteration was found in a study that showed that patients with urgency urinary incontinence had decreased abundance of Bacteroidetes compared to HCs (38). The patients suffering from renal transplantation and successive immunosuppressing therapy also had lowered levels of Bacteroidetes (34). Bacteroidetes can suppress enteric inflammation, suggesting that members of the phylum play a similar role in regulating the level of IL-8 in urinary tract system. Interestingly, there was a noticeable decrease in the phylum Bacteroidetes in newly diagnosed diabetics (39), and in our present study, we observed that patients with detectable IL-8 had T2DM for a longer duration than the NIL8 patients, suggesting that in the early state of diabetes, Bacteroidetes play a minor role in regulating the inflammatory process in diabetes.

We found that the genus of *Corynebacterium* in the urinary microbiota was enriched in WIL8 patients. A recent study reported that the prevalence of *Corynebacterium* correlated with concentrations of IL-6 and C-reaction protein in cancer patients (40). In our present study, the WIL8 group had a higher level of urine white blood cells, more leukocyte esterase and urine nitrite-positive cases, which indicated an inflammatory reaction was present in the urinary tract system, although no UTI was diagnosed currently. This might suggest that an inflammatory reaction and its correlation with urinary microbiota is established before a patient is diagnosed with UTI and the presence of obvious clinical manifestation, thus early detection of the interaction of the inflammatory response with urinary microbiota is valuable for clinicians to diagnose and treat UTIs in the early stages.

A higher abundance of *A. muciniphila* was correlated with a higher concentration of IL-8. Moreover, *Akkermansia* in the “≥HCs” group had a higher level of IL-8 than the “<HC” group. An *in vitro* model demonstrated that *A. muciniphila* could induce IL-8 production by enterocytes at cell concentrations 100-fold higher than those for *Escherichia coli* (41). Another study demonstrated that mice with low levels of inflammation were enriched for *Akkermansia* (42). Surprisingly, the patients from the WIL8 group had higher levels of urine leukocyte esterase and nitrite than the NIL8 patients, suggesting *Akkermansia* might play important roles in protecting patients away from UTIs, since patients in either WIL8 or NIL8 groups were not currently diagnosed with UTIs while they were recruited to the present study.

Lin et al. reported that the overgrowth of *Enterococcus* in diabetic mice was accompanied with increased IL-1β and TNF-α expression from Kupffer cells in intestine (43). Interestingly, our data indicated that *Enterococcus* was dramatically increased in the WIL8 group. Therefore, the abundance of *Enterococcus* might be regulating the level of cytokines in diabetic populations. Nienhouse et al. reported that *Pseudomonas* was enriched in positive urine culture specimens comparing to negative specimens. Also, *Pseudomonas* might cause the most severe inflammation which was accompanied by an increase in the number of inflammatory cells and IL-6 (44). This trend was similar to our study, in which patients with *Pseudomonas* in the “≥HCs” group had a higher level of IL-8. Interestingly, *Pseudomonas aeruginosa* were the predominant isolates of non-healing ulcers in diabetic foot patients (45), and diabetic foot infection has demonstrated higher concentrations of IL-6 and IL-1β than controls (46). Thus, *Pseudomonas* might be correlated to the inflammatory process in diabetic patients and might regulate the levels of cytokines. *Klebsiella*, being a recognized uropathogen (47), was enriched in female participants who developed UTIs after pelvic floor surgery (48). Interestingly, patients with *Klebsiella* in the “≥HCs” group had a higher concentration of IL-8. Huang et al. reported that diabetic patients was associated with relapse of recurrent bacteremia caused by *Klebsiella pneumonia* (49), suggesting that this bacteria is responsible for the inflammation process in diabetes. *S. anginosus* was also increased in the WIL8 group compared to the NIL8 group. A similar result was reported in the study conducted by Price et al., in which *S. anginosus* increased in subjects with UTIs compared to the non-UTI participants (50).

Members of the genus *Lactobacillus* exhibit probiotic effects in epithelial attachment, pathogen inhibition, and intestinal immunomodulation (51–53). However, their relative abundance was increased in the WIL8 group compared to the NIL8 group. Furthermore, *Lactobacillus* was one of the predictors of the presence of IL-8 in urine in our study. It was reported that *Lactobacillus* showed a rise in interstitial cystitis patients than HCs (54). However, a recent study reported that there were no associations between the presence of *Lactobacillus* and urinary cytokine levels in patients with interstitial cystitis (55). Therefore, *Lactobacillus* might play distinctive roles in regulating cytokine production in urine in different health statuses. *L. iners*, which may induce moderate IL-8 secretion and has moderate proinflammatory activity in the cervicovaginal bacterial community (16), was also increased in the WIL8 group. *L. mucosae* which has been demonstrated to possess IL-6 induction ability in macrophages (56), was found enriched in the WIL8 group. *L. reuteri* can suppress intestinal inflammation in a trinitrobenzene sulfonic acid-induced mouse colitis model *via* downregulation of gene expression of mucosal cytokine IL-6 and IL-1β in the colon (57). In another study, *L. reuteri* could produce molecules that had potential anti-TNF-α activity *in vitro* and antimicrobial compounds in diabetes (58, 59). Furthermore, intake of *L. reuteri* can increase insulin secretion, which might be due to augmented incretin release (60). Low-grade chronic inflammation is accepted as an internal metabolic adaptation pathway in T2DM, so the increased levels of members of the genus *Lactobacillus* might be believed to be players in controlling the development of inflammation in diabetes. Moreover, patients with the *Lactobacillus* “≥HCs” group had lower levels of IL-8 than the “<HCs” group, which also demonstrated that *Lactobacillus* contributed to inhibit the inflammatory process in the bladder.

In total, 18 bacterial genera contributed to the presence of IL-8 in the urine of T2DM patients. Most of these genera have not been reported by human urinary microbiota studies, except for *Actinomyces* (61) and *Lactobacillus* (47, 50, 61–65). Furthermore, very few of these genera have been reported in terms of their correlations with inflammatory cytokines. Interestingly, the abundance of *Ruminococcus* was positively correlated with the concentration of urinary IL-8, and can be considered as an important contributor to the presence of IL-8 as well. *Ruminococcus* spp. could induce dominant effector *ex vivo* mesenteric lymph node T-helper 17 responses (66), which is correlated to inducing the expression of IL-8 (67). In addition, *Ruminococcus* was more abundant in diabetic mice, and correlated negatively with delayed diabetes onset age (68). Thus, *Ruminococcus* might play a role in diabetes development by regulating the level of cytokines. *Actinomyces* spp. has been demonstrated to induce inflammatory cytokines by researchers (69), which were linked to the presence of urinary IL-8 in the present study suggesting therapy for inflammatory development in diabetes involving urinary microbiota should take a considerable to the 18 bacteria included in multiple analysis model.

There were several limitations in our study. First, the sample size in the WIL8 and NIL8 groups was not equal, which might affect the reliability of the results. Second, although all participants were not currently diagnosed with UTIs, we could not completely rule out the influence caused by previous occurrence of UTIs since it takes 6 months for diabetic patients to revert to normal glomerular filtration rate trends after an infection is cured (70); this might affect the growth environment of urinary microbiota and urine IL-8.

## Conclusion

To our knowledge, this is the first study focused on the associations between urinary microbiota and concentrations of urine IL-8 in T2DM patients. The bacterial community showed differences in the WIL8 and the NIL8 groups, and the concentrations of IL-8 were different in the “≥HCs” group and “<HCs” group. Findings from this study indicated that urine IL-8 is interplayed with urinary microbiota among T2DM patients. Future studies should focus on how the urinary microbiota affects the inflammatory cytokine excretion in the urinary tract, which might be conducive to explore novel therapies to regulate inflammation in T2DM patients.

## Ethics Statement

Ethics Committee of the First Affiliated Hospital, School of Medicine, Zhejiang University approved the study (Reference Number: 295).

## Author Contributions

LL and FL conceived and designed the study. ZL generated the sequencing data. FL and SL collected the samples. FL and YC conducted urine cultures and the urinalysis. FL extracted the bacterial DNA. ZL and FL analyzed the data, carried out the computational analysis, interpreted the data, and drafted the manuscript.

## Conflict of Interest Statement

The authors declare that the research was conducted in the absence of any commercial or financial relationships that could be construed as a potential conflict of interest.

## Materials and Methods

### Protocol of DNA Isolation

The tube was placed in liquid nitrogen for 1 min, and transferred into a water bath at 65°C for 5 min, with vigorous mixing. This last process was repeated three times with a final bath for 30 min. 50 µL *Agencourt AMPure XP* (Beckman Coulter, USA) was added to 100 µL of the urine pellet, vortexed for 30 s, and incubated for 5 min at room temperature. The tube was placed into a magnetic separator for 5 min, and DNA was bound to magnetic beads which were drawn to the wall of the microcentrifuge tube. The supernatant was carefully removed without disrupting the magnetic beads. The sample was washed twice with 200 µL 80% ethanol for 30 s, being placed on a magnet separator between each washing. The purified DNA was eluted with 50 µL ddH_2_O for 1 min. The beads, now released from the DNA, were collected with the magnet. The DNA-containing supernatant was transferred to a clean tube.

Figure S1 Genus-level operational taxonomic units different between healthy controls (HCs) and type 2 diabetes mellitus (T2DM) patients. Welch’s *t*-test was used to compare the abundance at the bacterial genus level between HCs and T2DM patients. The different genera were assigned only to those presenting a minimum variation at a significant level [*p* (corrected) < 0.05]. H and Pt represent HCs and T2DM patients, respectively.

Figure S2 Species-level operational taxonomic units different between NIL8 and WIL8 groups. STAMP software was used to calculate the species proportions in the two groups. Welch’s *t*-test was used to compare abundance at the species level for NIL8 and WIL8 specimens. The different species were assigned only to those presenting a minimum variation at a significant level [*p* (corrected) < 0.05].

Figure S3 Differences of *Lactobacillus* spp. between NIL8 and WIL8 groups. STAMP software was used to calculate the proportions of *Lactobacillus* spp. in NIL8 and WIL8 groups. Welch’s *t*-test was used to compare abundance of *Lactobacillus* spp. level for NIL8 and WIL8 specimens. The different levels were assigned only to those presenting a minimum variation at a significant level [*p* (corrected) < 0.05].

Figure S4 Correlation between the relative abundance of *Ruminococcus* and the concentration of urinary IL-8. A correlation analysis was carried out and a significance level of *p* < 0.05 was used.
