# Supplementary material for: Dysbiosis of the Urinary Microbiota Associated With Urine Levels of Proinflammatory Chemokine Interleukin-8 in Female Type 2 Diabetic Patients
Source: Front Immunol. 2017 Aug 25;8:1032. doi: 10.3389/fimmu.2017.01032 (PMC5603796; doi:10.3389/fimmu.2017.01032)
Supplement: Supplementary file 1 [file table_1.docx]

**Table S1. Comparison of richness and diversity of urinary microbiota in NIL8 and WIL8 groups**

| **Parameter^a^** | **NIL8** | **WIL8** | ***p* value** |
| --- | --- | --- | --- |
| No. of OTUs^b^ | 1744.71±1735.74 | 1689.54±975.14 | 0.865 |
| ACE^c^ | 3721.87±2245.53 | 3946.19±2430.47 | 0.708 |
| Chao1 | 3521.25±2131.67 | 3559.97±2150.51 | 0.943 |
| Shannon | 4.22±2.43 | 4.12±2.20 | 0.865 |
| Simpson | 0.64±0.24 | 0.68±0.26 | 0.593 |

^a^Parameters were calculated by QIIME software.

^b^The operational taxonomic units (OTUs) were defined at the 97% similarity level.

^c^ACE indicates Abundance-based Coverage Estimator.
