# Supplementary figures and images for: Dysbiosis of the Urinary Microbiota Associated With Urine Levels of Proinflammatory Chemokine Interleukin-8 in Female Type 2 Diabetic Patients
Source: Front Immunol. 2017 Aug 25;8:1032. doi: 10.3389/fimmu.2017.01032 (PMC5603796; doi:10.3389/fimmu.2017.01032)

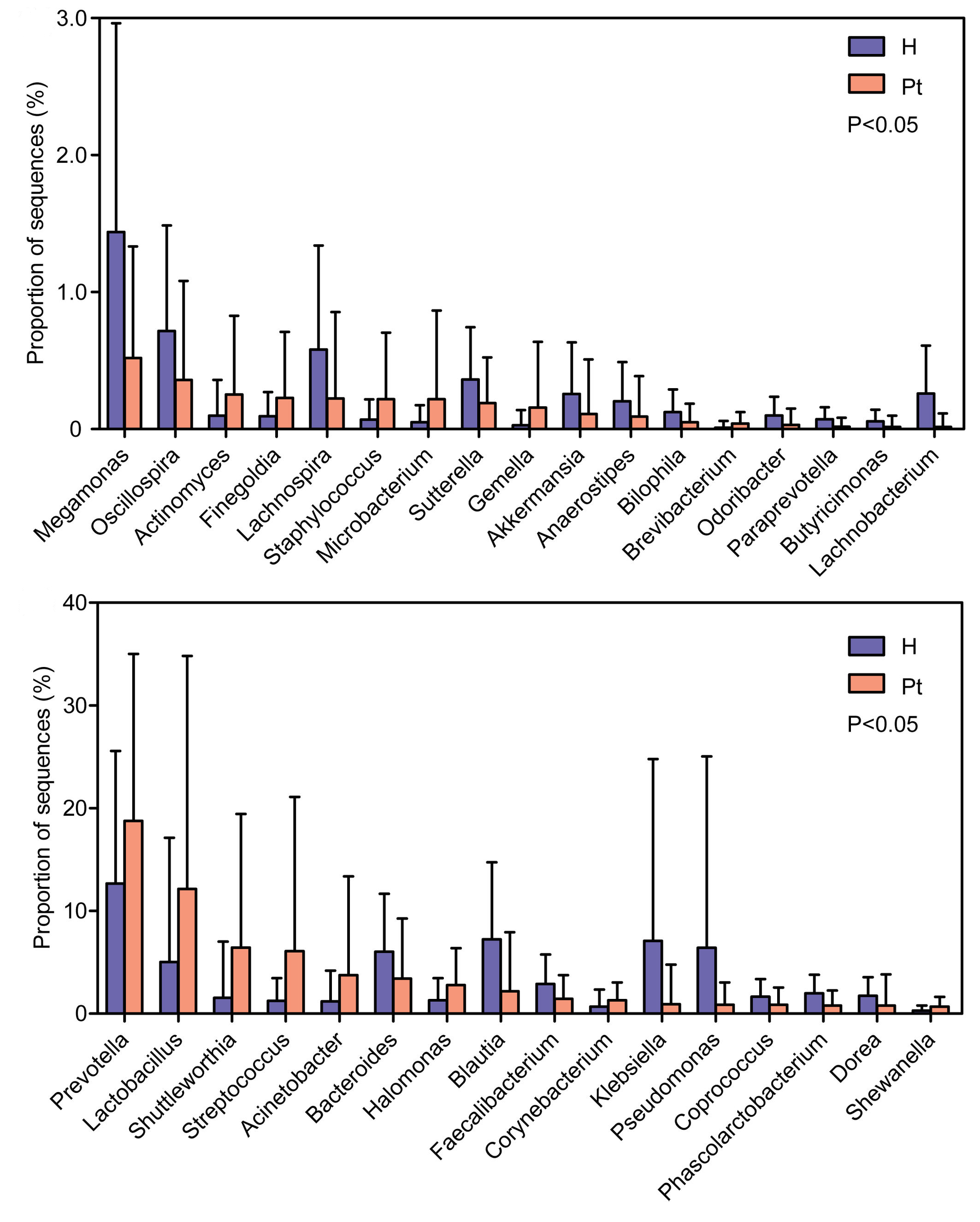

Supplement: Figure S1 — Genus-level operational taxonomic units different between healthy controls (HCs) and type 2 diabetes mellitus (T2DM) patients. Welch’s t-test was used to compare the abundance at the bacterial genus level between HCs and T2DM patients. The different genera were assigned only to those presenting a minimum variation at a significant level [p (corrected) < 0.05]. H and Pt represent HCs and T2DM patients, respectively. [file image_1.tif]

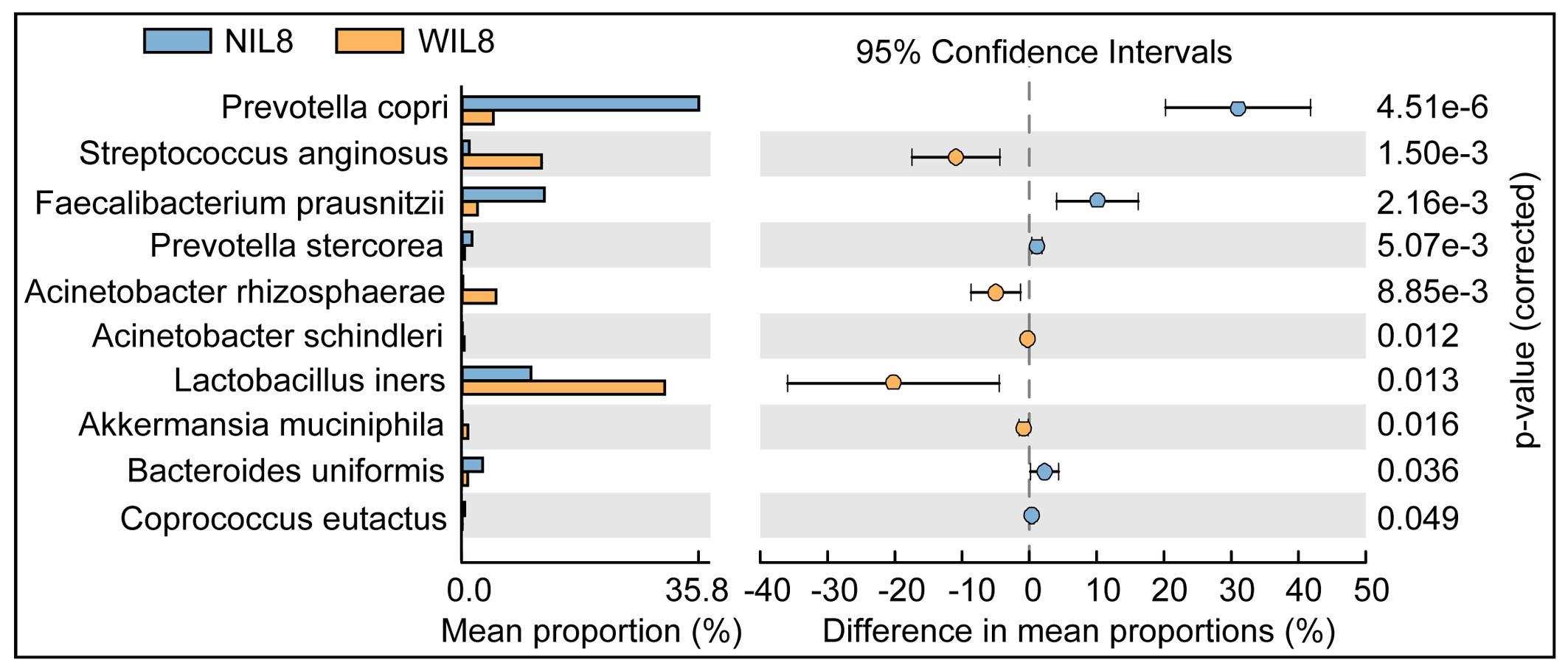

Supplement: Figure S2 — Species-level operational taxonomic units different between NIL8 and WIL8 groups. STAMP software was used to calculate the species proportions in the two groups. Welch’s t-test was used to compare abundance at the species level for NIL8 and WIL8 specimens. The different species were assigned only to those presenting a minimum variation at a significant level [p (corrected) < 0.05]. [file image_2.tif]

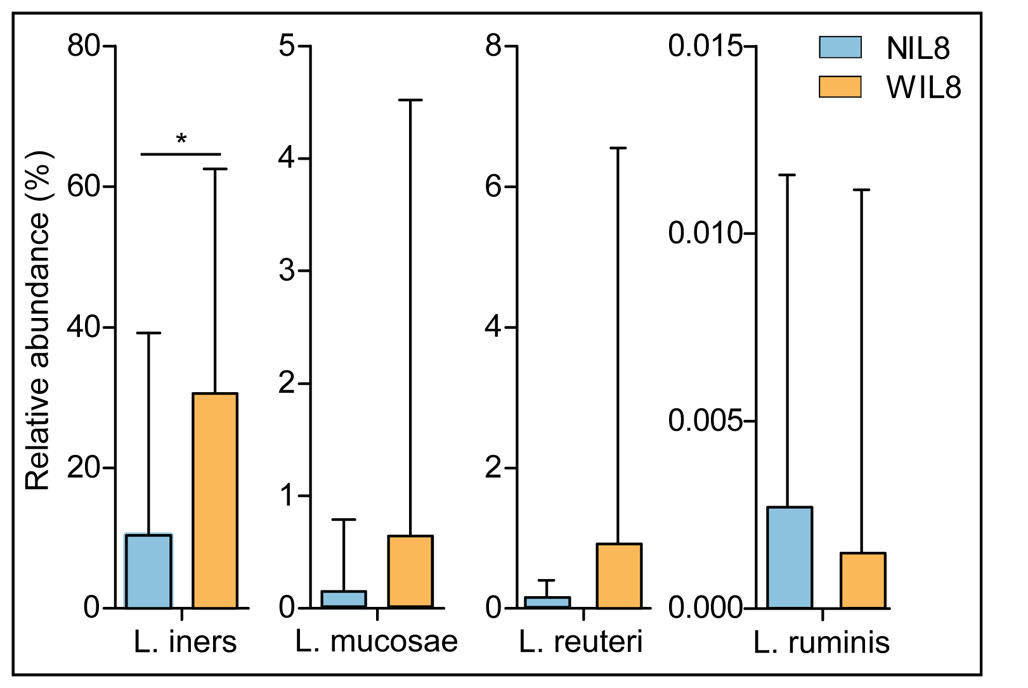

Supplement: Figure S3 — Differences of Lactobacillus spp. between NIL8 and WIL8 groups. STAMP software was used to calculate the proportions of Lactobacillus spp. in NIL8 and WIL8 groups. Welch’s t-test was used to compare abundance of Lactobacillus spp. level for NIL8 and WIL8 specimens. The different levels were assigned only to those presenting a minimum variation at a significant level [p (corrected) < 0.05]. [file image_3.tif]

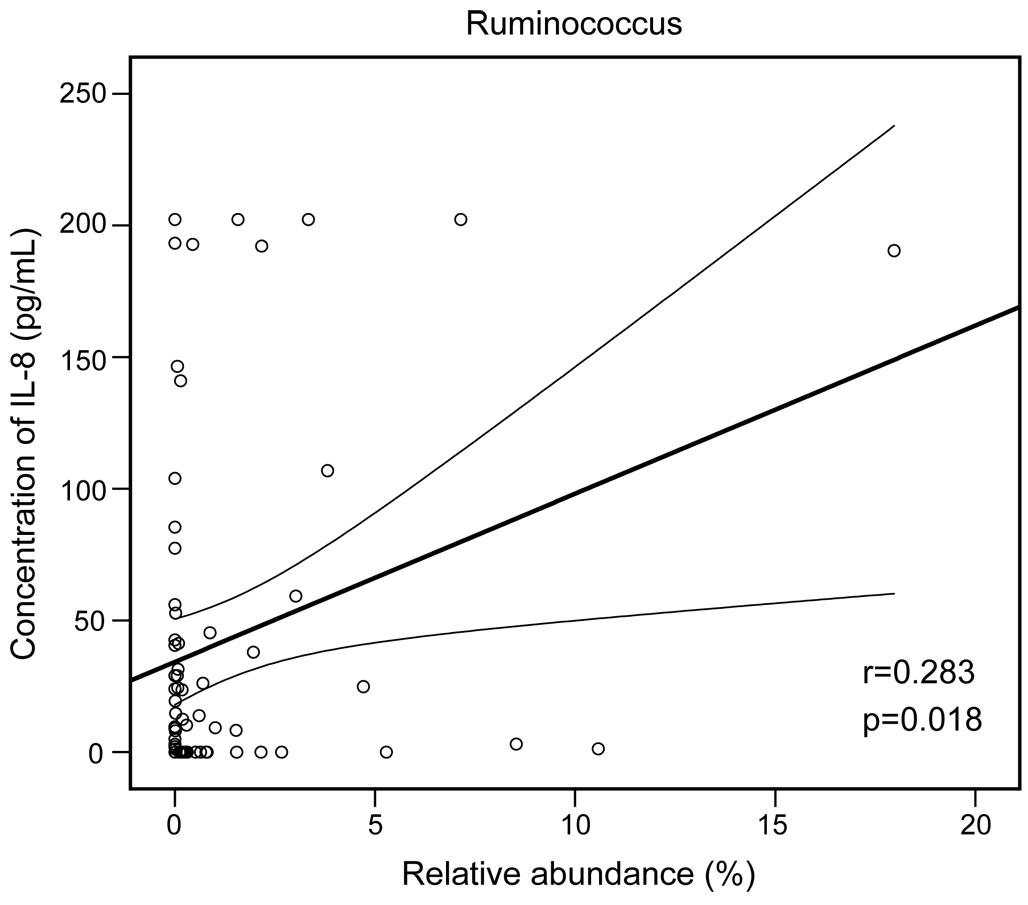

Supplement: Figure S4 — Correlation between the relative abundance of Ruminococcus and the concentration of urinary IL-8. A correlation analysis was carried out and a significance level of p < 0.05 was used. [file image_4.tif]
